# A Gutsy Move for Cell-Based Regenerative Medicine in Parkinson’s Disease: Targeting the Gut Microbiome to Sequester Inflammation and Neurotoxicity

**DOI:** 10.1007/s12015-019-09906-2

**Published:** 2019-07-17

**Authors:** Jea-Young Lee, Julian P. Tuazon, Sydney Corey, Brooke Bonsack, Sandra Acosta, Jared Ehrhart, Paul R. Sanberg, Cesario V. Borlongan

**Affiliations:** 1 Center of Excellence for Aging and Brain Repair, Morsani College of Medicine, University of South Florida, 12901 Bruce B. Downs Blvd. MDC 78, Tampa, FL 33612 USA; 2 Department of Neurosurgery and Brain Repair, Morsani College of Medicine, University of South Florida, 12901 Bruce B. Downs Blvd. MDC 78, Tampa, FL 33612 USA; 3 Saneron CCEL Therapeutics, Inc., Tampa, FL 33618 USA; 4 Department of Pathology and Cell Biology, Morsani College of Medicine, University of South Florida, Tampa, FL 33612 USA; 5 Department of Psychiatry, Morsani College of Medicine, University of South Florida, Tampa, FL 33612 USA

**Keywords:** Neurodegeneration, Cord blood stem cells, Plasma, Brain repair, Gastrointestinal mucosa

## Abstract

Pharmaceuticals and cell-based regenerative medicine for Parkinson’s disease (PD) offer palliative relief but do not arrest the disease progression. Cell therapy has emerged as an experimental treatment, but current cell sources such as human umbilical cord blood (hUCB) stem cells display only partial recapitulation of mature dopaminergic neuron phenotype and function. Nonetheless, stem cell grafts ameliorate PD-associated histological and behavioral deficits likely through stem cell graft-secreted therapeutic substances. We recently demonstrated the potential of hUCB-derived plasma in enhancing motor capabilities and gastrointestinal function, as well as preventing dopaminergic neuronal cell loss, in an 1-methyl-4-phenyl-1,2,3,6-tetrahydro-pyridine (MPTP) rodent model of PD. Recognizing the translational need to test in another PD model, we now examined here the effects of an intravenously transplanted combination of hUCB and plasma into the 6-hydroxydopamine (6-OHDA) lesioned adult rats. Animals received three separate doses of 4 × 10^6^ hUCB cells with plasma beginning at 7 days after stereotaxic 6-OHDA lesion, then behaviorally and immunohistochemically evaluated over 56 days post-lesion. Whereas vehicle-treated lesioned animals exhibited the typical 6-OHDA neurobehavioral symptoms, hUCB and plasma-treated lesioned animals showed significant attenuation of motor function, gut motility, and nigral dopaminergic neuronal survival, combined with diminished pro-inflammatory microbiomes not only in the nigra, but also in the gut. Altogether these data support a regenerative medicine approach for PD by sequestering inflammation and neurotoxicity through correction of gut dysbiosis.

## Introduction

Parkinson’s disease (PD) is the second most common neurodegenerative disorder especially in older individuals [1]. Traditionally, PD can be identified by Lewy bodies, which are intracellular collections of α-synuclein, and by the loss of dopaminergic neurons in the nigrostriatal pathway [2]. Freezing of gait, bradykinesia, tremors, and decreased balance are the cardinal symptoms of PD [1]. Morbidity is also intensified by other symptoms, in particular gastrointestinal problems and non-motor impairments [1, 3]. Although there have been many advances in this field, PD patients only receive palliative treatments. The long-standing gold standard of treatment is L-3,4-dihydroxyphenylalanine (L-DOPA), but the drug does not slow PD’s course. To the contrary, L-DOPA can cause adverse effects, such as dyskinesia, if used long-term. A small portion of patients have experienced symptom relief after deep brain stimulation [1, 3, 4]. Furthermore, cell-based therapies have been explored in the clinic [5, 6], but the results are often mixed, including some negative results [7]. Large-scale clinical application of fetal dopaminergic cell transplants is hampered by ethical and logistical problems [8, 9], necessitating the need for an alternative source of transplantable dopaminergic cells.

Almost three decades ago, the first attempted fetal derived dopaminergic cell transplant for human PD patients yielded the successful integration of the cells into the host’s dopaminergic network [9, 10]. While the transplant itself was successful, it resulted in only modest, gradually decreasing positive effects over the long-term [5, 6]. In fact, worsened dyskinesia was observed in some patients [11]. As a result, other options for cell transplant are being considered that may enhance the therapeutic benefits. Stem cell transplants promise to produce multi-pronged regenerative mechanisms called by-stander effects as an alternative brain repair processes beyond dopaminergic cell replacement [12, 13]. Among the many by-stander effects, anti-inflammation has been widely implicated as a key mechanism of action of stem cells [14]. In parallel, inflammation rampantly plagues many neurological disorders in their progression, and may even serve as a disease trigger. That an inflammation-mediated crosstalk exists between stem cells and brain disorders prompted us to investigate cell-based regenerative medicine for its direct anti-inflammatory property. Of utmost interest, our hypothesis advanced the notion that inflammation transgresses centrally, as well as peripherally, thus our proposed approach embodied sequestration of inflammation in the brain and the gut microbiome. Indeed, accumulating evidence has alluded to a pathological link between PD and gut microbiome [15–22]. The innovation here is that PD pathology may be affected by a non-CNS and non-dopaminergic organ [17, 19, 23] remote from substantia nigra, the conventionally targeted brain region of interest for PD.

Human umbilical cord blood (hUCB) cells represent a population of therapeutically active transplantable cells that have been employed for many neurological disorders. In 1988, juvenile Fanconi’s anemia was treated by infusing cord blood [24]. The therapeutic effects of cord blood cell transplants have been explored in the laboratory, with some reaching clinical trials, including disease indications for pediatric neurological disorders ranging from autism spectrum disorder, to cerebral palsy, peroxisomal or lysosomal storage disease [25–28], and adult neurodegenerative disorders such as PD, ischemic stroke, amyotrophic lateral sclerosis, traumatic brain injury, and Alzheimer’s disease [29–36].

Plasma and other components of hUCB have long been regarded as waste products [37], allowing easily accessible collection from the cell isolation process. In vitro cultures of human dental cells, hUCB-derived mesenchymal stem cells (MSCs), hUCB-derived T-lymphocytes, and human endothelial colony forming cells display improved cell viability from supplementation with cord blood plasma (CBP) [38–41]. CBP is composed of a distinctive cytokine profile with low concetrations of several pro-inflammatory cytokines, as well as high amounts of growth factors [29, 38, 42, 43]. The view that CBP may confer equally potent therapeutic effects as hUCB is bolstered by the observations that anti-inflammation and neuroprotection afforded by hUCB cell transplantation [32, 44–47] is achieved by CBP infusion in experimental stroke model [43, 48]. Our recent study shows that intravenous hUCB-derived plasma administration into the 1-methyl-4-phenyl-1,2,3,6-tetrahydro-pyridine (MPTP)-induced rat PD model ameliorates motor function and improves survival of dopaminergic neurons while reducing pro-inflammatory cytokines in the substantia nigra pars compacta (SNpc) [49]. Interestingly, such transplantation of hUCB plasma enhances colonic motility and gastrointestinal transit, coinciding with dampened inflammatory, harmful microbiota species in the gut [49].

The present study was designed as a translational approach [50, 51] to further probe the brain-gut hypothesis in another model of PD to complement our previous MPTP model. Here, we investigated the therapeutic effects of combined intravenous (iv) delivery of hUCB and CBP (hUCB+P) in the 6-hydroxydopamine (6-OHDA) lesion rat model of PD. Specifically, hUCB+P therapy was provided over a 7-day period starting at 7 days after intracranial (ic) stereotaxic administration of 6-OHDA. Neurological, motor, and non-motor symptoms were measured at days 14, 28, 42, and 56, with brain and gut tissues subsequently analyzed to reveal whether hUCB+P therapy promoted functional recovery from PD symptoms via sequestration of neurotoxicity and inflammation in both brain and gut.

## Methods

### Human Umbilical Cord Blood Cells and Plasma\

Saneron CCEL Therapeutics, Inc. supplied the frozen human umbilical cord blood cells and plasma (hUCB+P). The Sepax 2 full-automated cell processing system (Biosafe America Inc., Houston, TX) separated the cord blood cells and plasma for use. Before freezing, the BacT/ALERT Microbial detection system (bioMérieux, Durham, NC) was used to examine the cord blood cells and plasma units as aseptic. The human umbilical cord blood (hUCB) units were purchased commercially from GenCure (TCBB, West San Antonio, Texas) by Saneron CCEL Therapeutics, Inc. for research purposes under USF IRB# 131111. The de-identified cord blood units were processed by Saneron CCEL Therapeutics, Inc. using the Sepax 2 full-automated cell processing system (Biosafe America Inc., Houston, TX) which allowed for the sterile collection of both cord blood cells and plasma. Results for infectious disease testing of maternal blood samples, collected shortly after birth, was provided by GenCure, for infectious disease markers of HIV, hepatitis B and C, syphilis, CMV, and HTLV I&II. Each cord blood unit in the study was negative for all infectious disease markers. Saneron CCEL Therapeutics, Inc. supplied all of the processed, cryopreserved, human umbilical cord blood cells and plasma that was used in this study.

### Animal Conditions

The University of South Florida Institutional Animal Care and Use Committee (IACUC) approved the experimental protocol of this study. Normal environmental conditions (12-h light/dark cycle, 20 °C, and 50% relative humidity) were employed for the 8-week old male Sprague-Dawley rats (*n* = 32). For the duration of the 2-month study period, the rats were monitored on a daily basis, and the required steps were taken to limit the animal’s distress. The animals were fed with standard laboratory animal died (Teklad, Envigo USA). The personnel involved in the studies were blinded to the treatment state of the animals.

### Animal Model Preparation

Sodium pentobarbital (60 mg/kg, i.p.) was used to anesthetize the animal before positioning them in a Kopf stereotaxic frame. The Sprague-Dawley rats received an injection of 8 μg of 6-OHDA (Sigma Aldrich, St. Louis, MO) diluted in 3 μl sterile saline into the left substantia nigra pars compacta (SNpc; AP -5.0, ML +1.5, DV -8.0). This solution was injected for 4 min, and the needle was removed following an additional 5 min. The purpose of this 6-OHDA injection was to promote nigrostriatal dopaminergic neuronal degeneration. Sterility was maintained throughout the procedures. The rats were divided into groups: vehicle (*n* = 8) and hUCB+P (*n* = 24) treatment. At the start of the study, the weight of each animal was reported and checked for the next 7 days following the 6-OHDA lesion. 4 × 10^6^ human umbilical cord blood cells in 500 μL of cord blood plasma (hUCB+P) were intravenously injected using the jugular vein to the treatment group on the 7th day following insult. The vehicle group received an intravenous injection (iv) of PBS (500 μL). Following the first injection the animals received repeated administration at both 2 days and 5 days. On days 28, 42, and 56, tissue of the treated 6-OHDA rats was collected with 8 animals for each point in time.

### Behavioral Testing

Before the final hUCB+P treatment and at 14, 28, 42, and 56 days after, motor and non-motor behavioral testing was performed to assess common symptoms of Parkinson’s disease in the 6-OHDA model. Elevated body swing testing (EBST), beam walk, apomorphine-induced contralateral rotation testing, and rotarod comprised the standard behavioral measures to evaluate unilateral motor capabilities and neurological deficits as detailed elsewhere [12]. As described below, tests for sensitive non-motor deficits of PD included gastric emptying and colonic propulsion to examine changes in the gut environment.

### Distal Colonic Propulsion

A 5-mm-diameter glass bead was placed approximately 2 cm from each rat’s anus into the distal colon on days 14, 28, 42, and 56 following hUCB+P injection to evaluate colonic propulsion. Each animal was then placed back in their cages without food or water following insertion of the glass bead. The rats were closely monitored to observe any abnormal changes in behavior. The time of glass bead excretion, called mean expulsion time (MET), was recorded for each individual animal to the nearest 1.0 s. The increase in MET versus that of vehicle control rats was determined to measure colonic propulsion.

### Gastric Emptying

A solution of charcoal (10%) was administered orally to the animals at certain time points, as well as acacia gum (2%) via gavage. The rats were then placed back in their cages for 30 min prior to euthanizing by carbon dioxide. Each animal’s intestines were harvested, and the length charcoal meal moved was recorded. The distance from pylorus through the anus was measured for each animal to determine the complete length of the intestine. The data is displayed as a percent, with distance traveled by the charcoal meal in relation to the entire intestine length.

### Tissue Collection

At 28, 42, and 56 days following hUCB+P injection, the animal groups (*n* = 8) were deeply anesthetized and sacrificed. 200 mL of cold phosphate buffer saline (PBS) and then 200 mL of 4% paraformaldehyde in PBS was used for transcardial perfusion. The brain and intestines were harvested from each animal. These tissues were fixed in additional paraformaldehyde solution at 4 °C for 24 h, and then replaced by 30% sucrose in phosphate buffered saline (PBS) for at least 16 h before sectioning. A cryostat was used to coronally section the tissues at 40 μm. The sections were collected in cryoprotectant solution and stored at −20 °C.

### Immunofluorescence

On every 6th coronal tissue section, immunofluorescent staining targeting the SNpc was performed for tumor necrosis factor (TNF), tyrosine hydroxylase (TH), and major histocompatibility complex II (OX-6). The sections were anatomically compared and matched across all animals. 0.1 M phosphate-buffered saline (PBS) was used to wash eight free-floating sections from each animal to eliminate any quantity of cryoprotectant solution. A blocking solution of PBS supplemented with 10% normal horse serum and 0.1% Triton X-100 was used to store the tissues for 1 h. A dilution of mouse anti-rat TH (1:4000 tyrosine hydroxylase; 22941; Immunostar, Hudson, WI), mouse anti-rat MHC II (anti-RT1B (OX-6); NB100–65541; Novus Biologicals, Centennial, CO), or rabbit anti-rat TNF-α (ab6671; Abcam; Cambridge, MA) antibodies in PBS supplemented with 10% normal goat serum and 0.1% Triton X-100 was used to incubate the sections overnight at 4 °C. Normal goat serum was obtained from Vector Laboratories (Cat # S1000, Burlingame, CA, USA), while normal horse serum was obtained from Vector Laboratories (Cat # S2000, Burlingame, CA, USA). The coronal sections were washed in PBS three times for 10 min, and then stored for 90 min in PBS supplement with 10% normal goat serum and 0.1% Triton X-100 containing the corresponding secondary antibodies, goat anti-mouse IgG-Alexa 488 (green) (1:500; Invitrogen) and goat anti-rabbit IgG-Alexa 594 (red) (1:750; Invitrogen). Following this incubation, PBS was used to wash the sections five times for 10 min. Fluoromount (Sigma; F4680) was the mounting medium for cover-slipping. The tissues were examined using independent channels within an Olympus FV1000 laser scanning confocal microscope set with Fluoview SV1000 imaging software. Image analysis of positive staining was determined using NIH ImageJ software (version 1.46). 3% normal goat serum in PBS replaced the primary antibodies in the controls. These controls displayed no immunoreactivity.

### Gut Microbiome Analysis

The gut microbiome was examined using the following measures to determine the prevalence of particular inflammatory, possibly harmful, bacterial species. Specific nucleotide sequences are provided in Table 1. The rat’s fecal microbiota was analyzed and identified using fluorescent in-situ hybridization (FISH). 1 g of collected feces was suspended in PBS (2 mL). Repetitive pipetting was performed to homogenize the fecal matter, followed by mixing with a vortex and addition of 6 mL of 4% paraformaldehyde for fixation. Following an overnight incubation period at 4 °C, the fecal tubes were centrifuged for 5 min at 10 g to sediment the undigested matter. The supernatant was placed in a new tube and centrifuged an additional time. The supernatant was then transferred to a new tube, and centrifugation was repeated for 5 min at 60 g. The supernatant was removed, and the resulting bacterial pellet was suspended in 500 μL of hybridization solution including 50% (*v*/v) formamide, 100 μg/mL salmon sperm DNA, 5x saline-sodium citrate buffer, and 0.1% (v/v) Tween 20. The samples were incubated for 30 min 37 °C. 50 μL of each sample were then added into a new tube with 2.7 μL of fluorescently labeled oligonucleotides. Following an incubation period at 45 °C for 2.5 h in the dark, the tubes were centrifuged at 60 g for 5 min. The supernatant was removed and discarded, and the pellet was resuspended in 20 μL of 0.1 M sterile sodium chloride. This washing was repeated 2 additional times, and the pellets were then resuspended in 20 μL of PBS. The samples were centrifuged at 13,000 g for 5 min. 10 μL was transferred to a new slide sterilized with EtOH. 5 μL of vectashield mounting medium with DAPI was added to the slides and cover-slipped. An Olympus FV1000 laser scanning confocal microscope with Fluoview SV1000 imaging software was used to collect the images at 40x.

**Table 1:** Specific nucleotide sequences

probe	Sequence	Specificity	Reference
BAC303	5’-CCAATGTGGGGGACCTT-3’	*Bacteroides-Prevotella* group	Manz et al. 1996 [52]
EREC482	5’-GCTTCTTAGTCAGGTACCG-3’	Clostridium cluster XIVa	Franks et al. 1998 [53]
LAB158	5’-GGTATTAGCA(C/T)CTGTTTCCA-3’	*Lactobacillus-Enterococcus* group	Harmsen et al. 1999 [54]

### Statistical Analysis

Behavioral results, image data, and microbiome analysis are expressed as mean ± S.D. Statview (Abacus Corporation) was used to perform these statistical analyses. The study results were evaluated using one-way ANOVA with Bonferroni post-hoc analysis. A significant *p* value was determined to be *p* < 0.05.

## Results

### hUCB+P Attenuates Motor Deficits in the 6-OHDA Model of PD

**Fig. 1:**
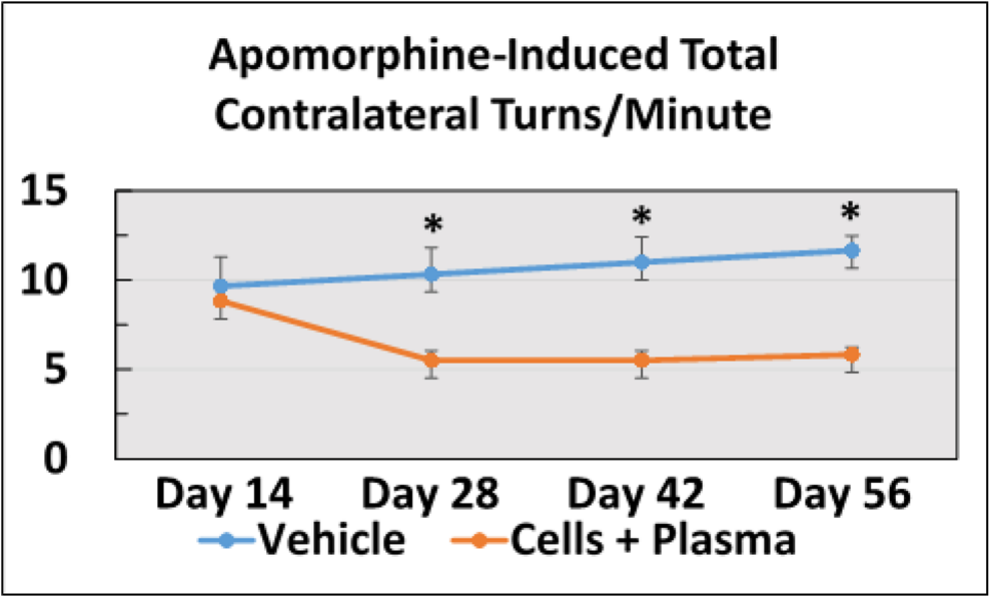
Administrations of cord blood cells with plasma in the 6-OHDA PD rat model: Assessment of 6-OHDA induced neurological deficits. Asymmetrical motor behavior was assessed using apomorphine-induced rotational behavior post-treatment on Day 14, 28, 42, and 56. Stem cell administration significantly reduced the total number of apomorphine-induced contralateral turns starting at day 28 and persisted up to day 56 post-lesion. (* *p* < 0.001)

**Fig. 2:**
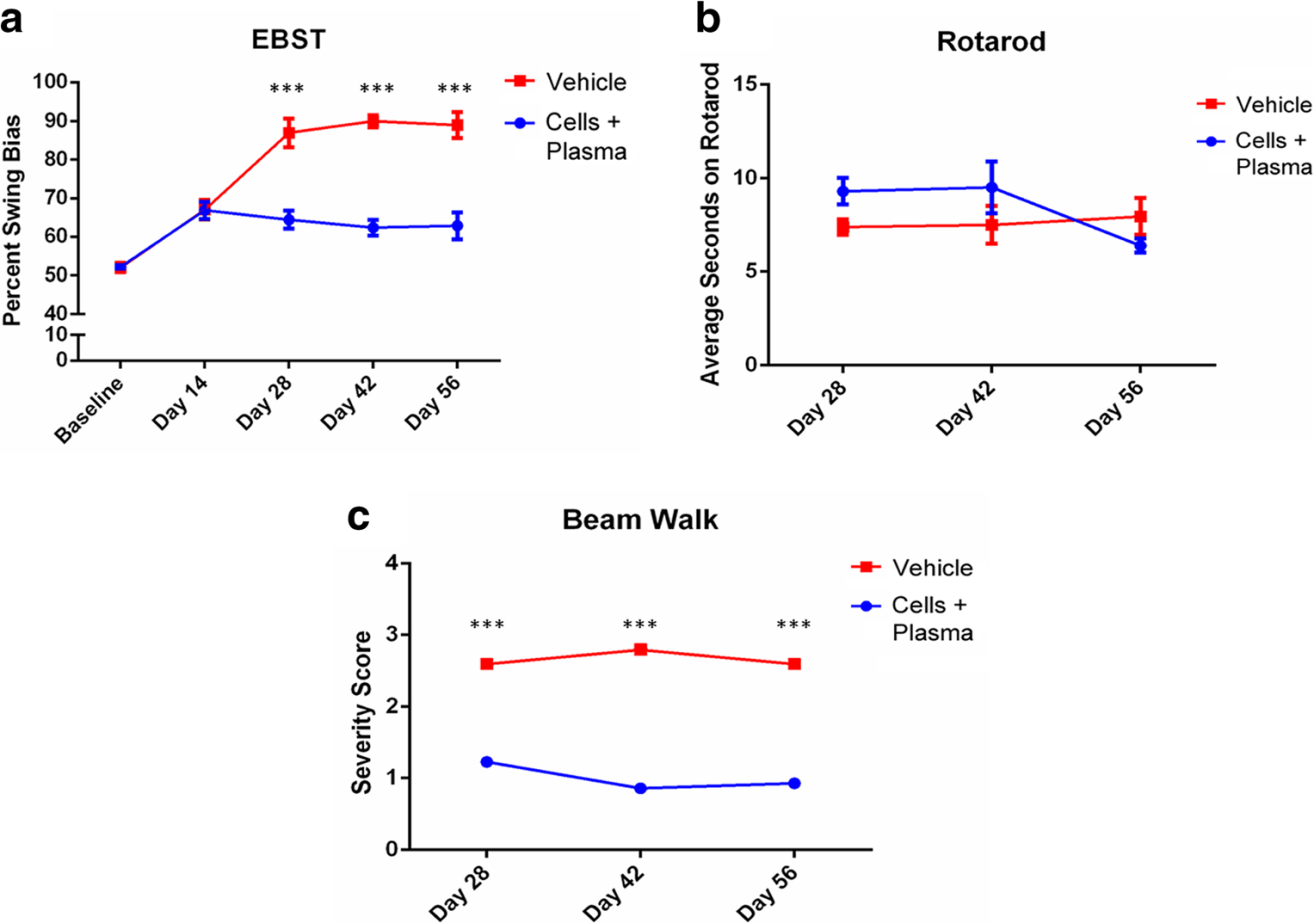
Administrations of cord blood cells with plasma in the 6-OHDA PD rat model: Assessment of 6-OHDA induced neurological deficits (**a**). Neurological function was also assessed using the elevated body swing test at Day 14, 28, 42, and 56 post treatment. Animals receiving stem cells exhibited a significant reduction in swing bias observed on Days 28, 42, and 56. (*** p < 0.001). Administrations of cord blood cells with plasma in the 6-OHDA PD rat model: Assessment of motor function improvement (**b**). Motor coordination and balance were assessed post-treatment on Day 28, 42, and 56. Improvements in motor coordination were observed at 28 days with combined cord blood cell and plasma treatment, but not at the later time points (42 and 56 days). Administrations of cord blood cells with plasma in the 6-OHDA PD rat model: Assessment of locomotor improvements (**c**). Locomotor performance was assessed post-treatment on Day 28, 42, and 56. The combination of cord blood cells with plasma resulted in significant improvement in motor coordination at each time point tested (*** p < 0.001)

In this study, the efficacy of a combined cord blood cell and cord blood plasma (hUCB+P) therapy was investigated in the 6-OHDA lesion rat model of PD. There were no adverse reactions observed in any of the animals that received iv hUCB+P and none of the animals were withdrawn from the study before completion. Motor function was assessed in PD animals by observing both apomorphine-induced rotational behavior (Fig. 1) and elevated body swing testing (Fig. 2a). This motor testing was used to validate the induction of PD-like pathology resulting from unilateral stereotaxic injection of 6-OHDA, and to assess any potential therapeutic benefit from hUCB+P administration. The induction of rotational behavior and body swing bias observed in vehicle-treated animals indicated the establishment of PD-like neurological and motor symptoms that are consistent with PD progression. Lesioned animals treated with hUCB+P exhibited a significant reduction in total apomorphine-induced contralateral turns at the 28, 42, and 56 day time points compared to vehicle-treated lesioned animals (*p* < 0.05). Additionally, elevated body swing testing revealed a significant reduction in swing bias observed from lesioned animals that received hUCB+P versus the vehicle group. (*p* < 0.001). Motor coordination test using the Rotarod was employed to determine overall motor coordination in the study animals (Fig. 2b). Lesioned animals that received the hUCB+P after 6-OHDA showed improvement at the 28 day time point over vehicle-treated lesioned animals, although significance was not observed at the other time points assayed. Locomotor performance using the beam walk test (Fig. 2c) was also observed for all animal groups and time points in this study. Lesioned animals that received hUCB+P showed significantly improved beam walk scores when tested at Day 28, Day 42, and Day 56 after 6-OHDA induction (*p* < 0.001).

### hUCB+P Increases Colonic Motility, but does not Significantly Affect Gastrointestinal Transit

**Fig. 3:**
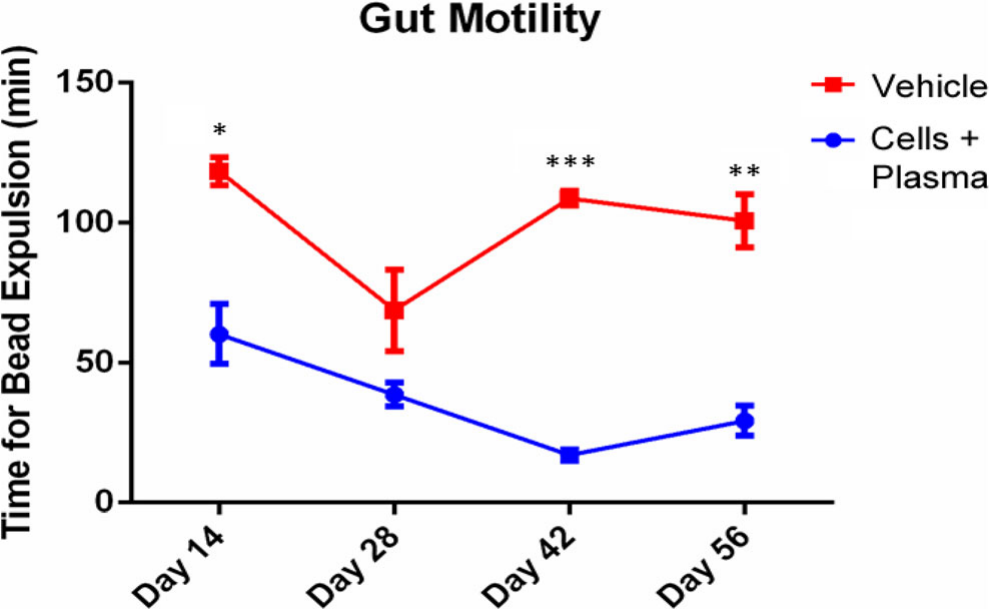
Administrations of cord blood cells with plasma decreases the latency of bead expulsion in the 6-OHDA PD rat model: Assessment of Gastrointestinal Function. Colonic motility was investigated post-treatment on Days 14, 28, 42 and 56. Bead expulsion latency analysis demonstrated that the 6-OHDA administration results in delayed expulsion of the bead in vehicle treated animals. Improvement in bead expulsion time was observed at all time points tested with significant decreases at Day 14 (*p* < 0.05), Day 42 (p < 0.001), and Day 56 (*p* < 0.01)

**Fig. 4:**
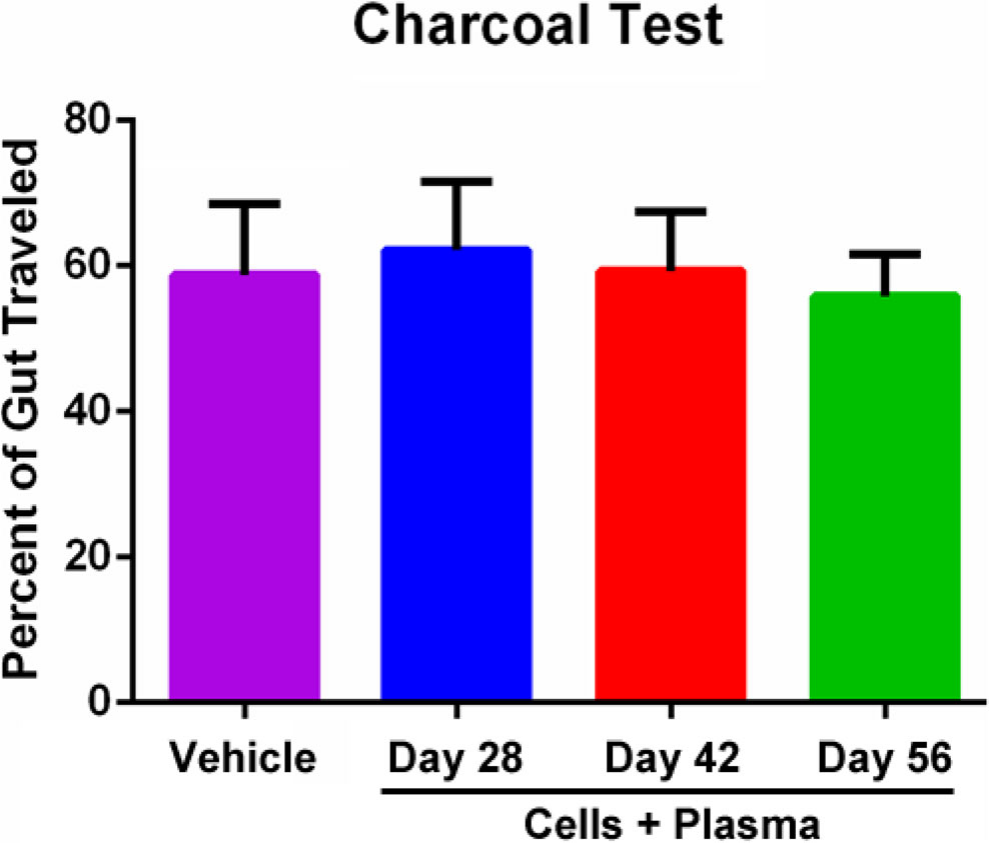
Administration of cord blood cells with plasma in the 6-OHDA PD rat model: Assessment of Gastrointestinal Function. Gastrointestinal (GI) transit (from the pylorus through the anus) was measured using charcoal meal testing on post-treatment Days 28, 42, and 56. Animals receiving cord blood cells and plasma showed that the GI transit at early time points (Day 28) resulted in a slightly greater distance traveled by the charcoal meal

To determine the efficacy of hUCB+P in reducing the parkinsonian symptoms related to the gastrointestinal (GI) system, colonic motility and GI transit tests were performed. Colonic motility was assessed by inserting a small glass bead into the animal’s rectum and measuring the amount of time needed to expel the bead (Fig. 3). Testing at all time points showed improved gut motility through decreased latency for bead expulsion in lesioned animals that received hUCB+P compared to vehicle-treated lesioned animals, reaching significance at Day 14 (*p* < 0.05), Day 42 (*p* < 0.001), and Day 56 (*p* < 0.01). Additionally, GI transit was observed by providing animals with a charcoal meal approximately 30 min prior to euthanasia (Fig. 4). During tissue collection the distance the charcoal meal traveled within the intestines was measured. A slight improvement in the distance traveled was observed at Day 28 in lesioned animals that received hUCB+P, but this change was not significant compared to vehicle-treated lesioned animals (*p* > 0.05).

### hUCB+P Improves Dopaminergic Neuronal Survival and Decreases Inflammation

**Fig. 5:**
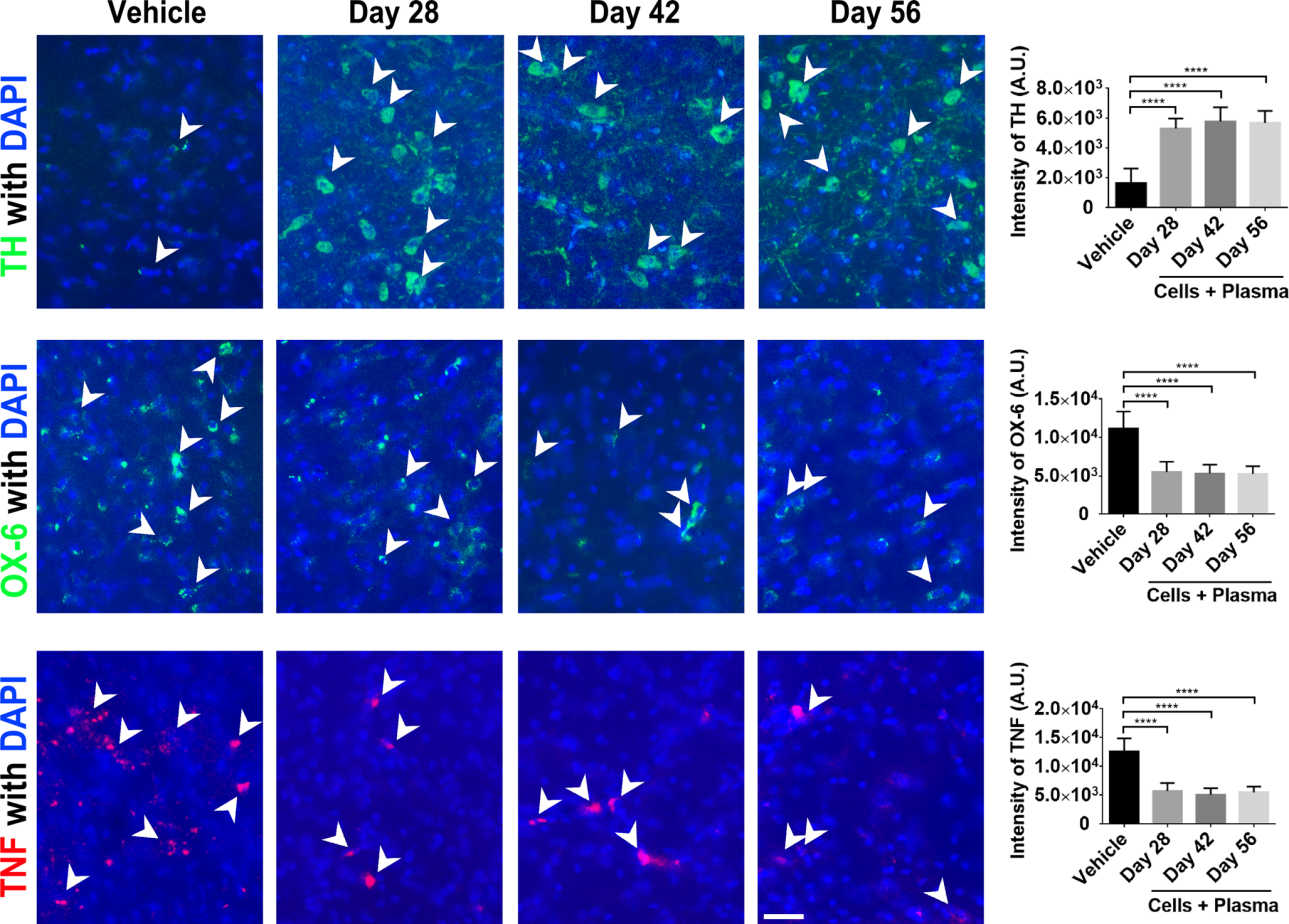
Administration of cord blood cells with plasma in the 6-OHDA PD rat model: Assessment of TH-positive dopaminergic neurons, immune cell activation, and pro-inflammatory cytokine production. Tyrosine hydroxylase (TH) staining (top row) was used to investigate the potential effect of combined cord blood cell with plasma injection on dopaminergic neuron populations in the substantia nigra pars compacta (SNpc). Analysis of fluorescent intensity revealed significant protection and retention of dopaminergic cells in the SNpc in 6-OHDA animals treated with cord blood cells and plasma, compared to vehicle controls. Cord blood treated groups also demonstrated significant reductions immune cell activation (OX-6) and pro-inflammatory cytokine production (TNF). Overall, the combination of cord blood cells and plasma can significantly modulate the exacerbated immune response through downregulation of pro-inflammatory cytokine production and immune cell activation in the 6-OHDA rat model at all time points tested. Photomicrographs correspond to representative SN in coronal sections immune-labeled with TH, OX-6 or TNF antibody. Scale bar = 50 μm

Histopathological examination of brain tissues was performed to document changes in dopaminergic neuron populations, immune cell activation, and the level of inflammation. 6-OHDA administration has been directly linked to the loss of dopaminergic neurons in the SNpc and resultant parkinsonian-like pathologies. Staining for the enzyme tyrosine hydroxylase (TH) is a common method used to characterize the extent of dopaminergic neuron loss due to this 6-OHDA insult (Fig. 5). Image analysis of TH staining revealed significant sparing of TH-positive cells in lesioned animals that received hUCB+P compared to vehicle-treated lesioned animals for all time points assayed (*p* < 0.0001). Interestingly, expression of both the OX-6 immune cell marker and the TNF pro-inflammatory cytokine in the SNpc was significantly decreased in lesioned animals that were injected with hUCB+P compared to vehicle-treated lesioned animals (p < 0.0001) (Fig. 5). These results indicate that hUCB+P attenuated 6-OHDA-induced neurotoxicity as evidenced by reduced dopaminergic neuron cell loss with a corresponding suppression of inflammation at least up to the 56-day study period.

### hUCB+P Reduces Intestinal Inflammatory Cytokine Production and Immune Response

**Fig. 6:**
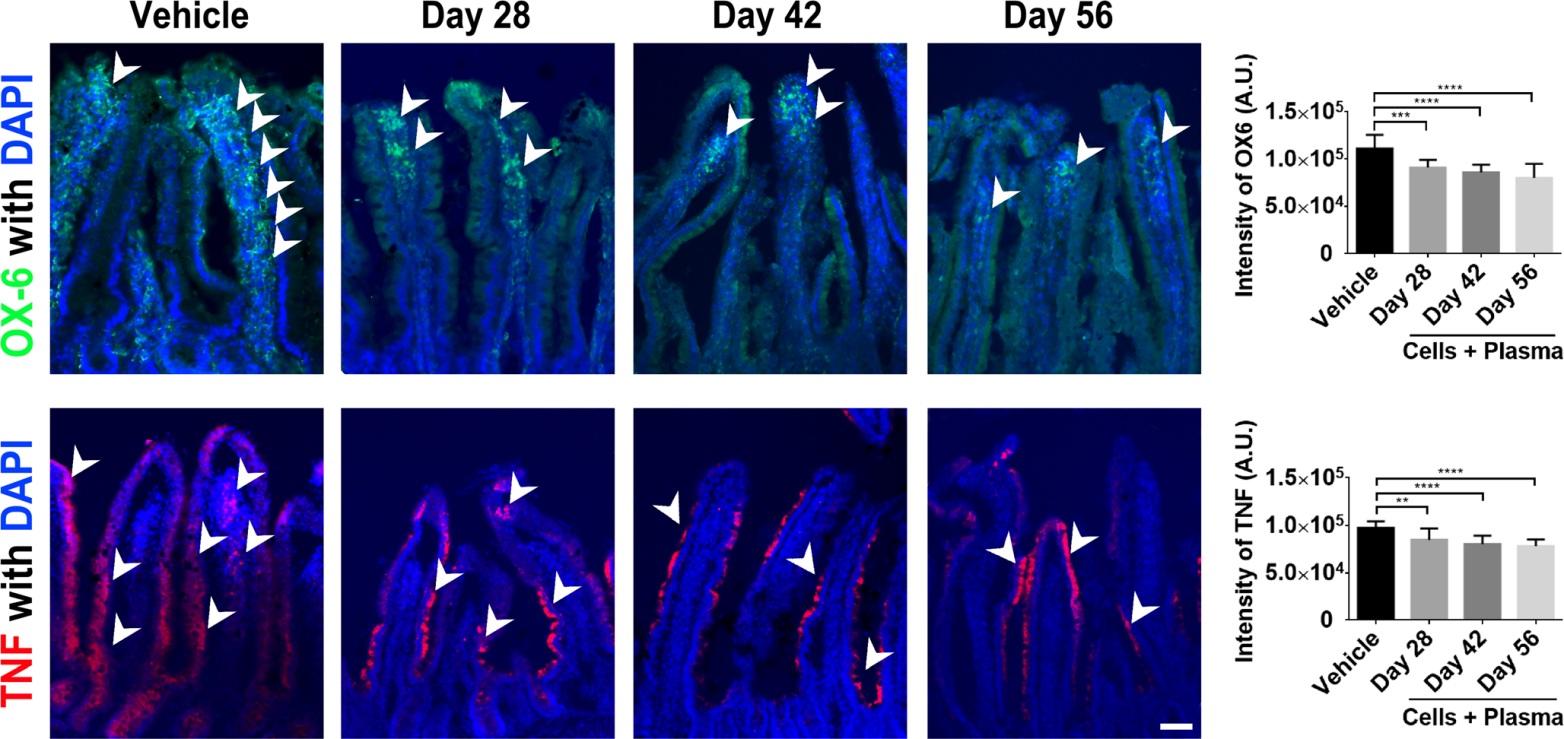
Administration of cord blood cells with plasma in the 6-OHDA PD rat model: Assessment of immune cell activation, and pro-inflammatory cytokine production in the intestine. Confocal imaging shows a significant reduction in OX-6 (MHC II) expression in the intestinal mucosa of 6-OHDA animals that were given a combination of cord blood cells and plasma. This therapy also resulted in a significant decrease in TNF expression in this animal model. Quantitative analyses of the estimated OX-6 expression and TNF are displayed in the graphs above. Representative merged images above show co-localization of OX-6 (Green) or TNF (Red) with DAPI+ (Blue) expression from cells in the small intestine of 6-OHDA PD animals. Arrow heads indicate positive staining of OX-6 and TNF expression in intestinal villi. Scale bar = 100 μm

6-OHDA induced changes in immune cell activation and production of the pro-inflammatory TNF cytokine was also examined in the intestinal tissues collected from study animals (Fig. 6). Similar to observations made with brain tissues, there was a significant reduction in both the expression of the OX-6 immune cell marker for Day 28 (*p* < 0.001), Day 42 (*p* < 0.0001) and Day 56 (p < 0.0001), as well as the pro-inflammatory cytokine TNF for Day 28 (*p* < 0.01), Day 42 (p < 0.0001) and Day 56 (p < 0.0001) in animals that were given hUCB+P compared to vehicle-treated lesioned animals. These findings demonstrate that iv administration of hUCB+P effectively reduced the 6-OHDA-mediated inflammation in the gut, suggesting that in this unique therapeutic combination robustly sequestered inflammation not just in central, but also peripheral tissues.

### hUCB+P Dampens the Amount of Inflammatory Microbiota in the Gut

**Fig. 7:**
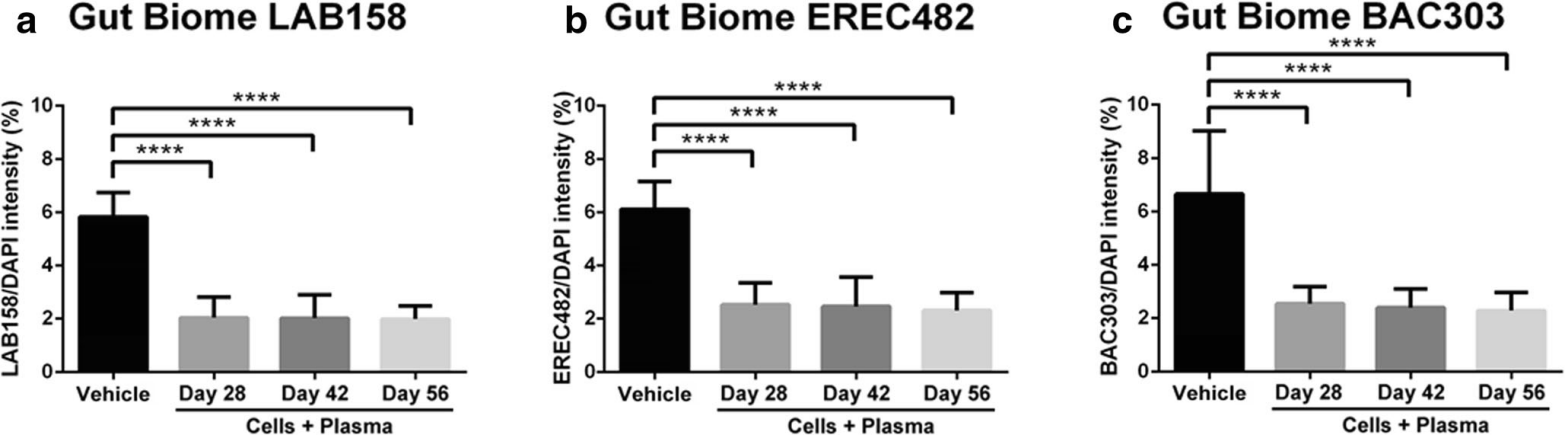
Administration of cord blood cells with plasma in the 6-OHDA PD rat model: Assessment of potentially harmful microbiotic species within the gut of 6-OHDA animals receiving cord blood therapy. Fluorescent in-situ hybridization (FISH) analysis was used to identify specific microbiota within the feces of rats that received 6-OHDA insult. Quantitative analysis revealed a significant reductions in the percentage of LAB158, BAC303, and EREC482 microbiota in the intestines of animals that were provided a therapy consisting of cord blood cells with plasma, compared to vehicle controls

That a dysbiotic gut may reflect a pathologic condition in the brain is likely to reveal the prevalence of deleterious microbiotic species in individuals affected by neurodegenerative disorders. In this study, the occurrence of potentially harmful microbiota species initially identified in the earlier proof-of-concept study [49] were investigated using fluorescent in-situ hybridization (FISH) (Fig. 7). Significant reduction in the amount of inflammation-linked microbiomes LAB158, BAC 303 and EREC482 were observed in the gut of lesioned animals that were given hUCB+P than those that received vehicle (*p* < 0.0001), suggesting that gut microbiomes stand as sensitive biomarkers of inflammation in PD.

## Discussion

The aim of this study was to assess whether hUCB+P treatment is a viable therapeutic combination for treating both motor and non-motor symptoms of the unilateral 6-OHDA lesion model of PD. The combined treatment’s efficacy was assessed behaviorally and histologically, and probed for the potential mechanistic role of the gut microbiome. The present results demonstrated that hUCB+P’s improved motor and non-motor functions, specifically gut motility, which were accompanied by dampened inflammatory-associated species of gut microbiota. Moreover, treatment with hUCB+P improved decreased levels of inflammatory gut microbiota, reduced the activation of immune cells (OX-6) and TNF-α in both the gut and brain tissues, and attenuated the dopaminergic (TH) cell loss in the SNpc. These therapeutic effects mimicked our earlier results that showed similar potentiation of PD symptoms, reduction of neurotoxicity, and suppression of deleterious inflammatory gut microbiomes in the MPTP model of PD [49].

Three innovative advances were shown here over our recent MPTP model for testing hUCB+P therapy [49]. First, because the unilateral 6-OHDA model allowed an exaggerated motor deficit to be manifested as evidenced by apomorphine-induced rotations and elevated body swing test, which were not possible with the MPTP bilateral lesions, we were able to capture the therapeutic effects of hUCB+P therapy on motor function more accurately. Second, the MPTP model resembles a more acute stage of PD, while 6-OHDA which produced at least >80% dopaminergic depletion in the SNpc approximated the chronic stage of PD. Accordingly, we demonstrated here that hUCB+P therapy was effective in ameliorating chronic PD symptoms in addition to our earlier observation of its benefits in the early phase of the disease. Third, relevant to the observed functional benefits of hUCB+P therapy in acute and chronic stages of the disease, we also found here that gut dysbiosis resulting from MPTP and 6-OHDA likely accompanied the onset and progression of the disease, and that this cell-based regenerative medicine effectively suppressed the aberrant inflammation that plagued the gut, which possibly translated to reduced neurotoxicity and improved therapeutic outcomes.

Cell replacement and by-stander effects account for many of regenerative mechanisms ascribed to cell therapy [14, 55]. In the past, the cell replacement mechanism was fundamental to the success of fetal dopaminergic cell transplants, which vastly improved parkinsonian behavioral and histological deficits in experimental animals, but only provided modest amelioration of symptoms in PD patients despite good graft cell survival and integration with the host tissue [5, 6, 9, 10]. In addition, worsening dyskinesias were experienced by some patients with fetal dopaminergic transplants [11]. Because of these mixed results, coupled with logistical and ethical concerns associated with the use of fetal tissues, alternative sources of transplantable cells, such as stem cells, have been explored. In part due to the advent of non-dopaminergic and even non-neural stem cells, cell replacement mechanism of brain repair has been replaced by by-stander effects [12, 13]. To date, although the by-standers effects, primarily through anti-inflammatory factors, have been widely examined in the brain, very few studies have ventured probing their potential actions peripherally [29], in particular the effects on the gut microbiome. Aberrant inflammation has been linked to exacerbation of dopaminergic cell depletions and worsened disease symptoms in animal models of PD [56–58], with auspicious contribution from the gut [17, 19, 59–61].

Some limitations of the study relate to the present FISH approach with our reliance on the intensity of the examined microbiomes. Parallel studies using other techniques in assessing gut microbiome (e.g. next generation sequencing) will be needed to confirm and fully quantify the levels of expression of these microbiomes. Moreover, functional targeting of microbiome requires assessment of outcome on the level of the biome and likely not single strains, and this warrants further investigations. Additionally, while we showed here that the PD lesion model and hUCB+P therapy alter the gut microbiome, the contribution of the animal diet will need ample consideration, and can be manipulated accordingly to reveal its potential effects on the resulting microbiome after a brain insult and stem cell transplantation. Finally, our study is limited to males and will require validation in female animals, in addition to other biological variables such as age and relevant PD risk factors, in order to fully capture the clinical relevance of our findings.

Recognizing that the gut represents a novel therapeutic target for PD, we envisioned that cell therapy with hUCB+P may sequester both neurotoxicity and inflammation-plagued gut microbiome. Here, we showed that hUCB+P may circumvent the need for an ample supply of dopaminergic cells for transplant, which has been a challenge for fetal tissues as well as more recent stem cell sources, such as embryonic stem cells and induced pluripotent stem cells [1, 62–64]. In addition, therapeutic investigations on PD designed to abrogate inflammation have focused on the neurodegenerative process within the nigrostriatal dopamine pathway concomitant with motor symptoms. The present study concedes the need to remain vigilant on the dopamine-mediated motor symptoms, but also sheds new light on the possibility of studying non-motor PD symptoms outside of the dopaminergic system and even beyond the brain, in line with accumulating evidence that these central dopaminergic systems work collaboratively with peripheral non-dopaminergic systems [3, 65–68]. To this end, we targeted the gut microbiome via hUCB+P therapy, providing critical evidence of a major role played by a non-CNS and non-dopaminergic organ in PD pathology and its treatment. Furthermore, this study offers additional evidence to previously observed by-stander effects in PD primate and rat models [12, 13, 65, 69–71], in that hUCB+P may enhance the therapeutic outcome by suppressing neurotoxicity via correction of gut dysbiosis [72]. Finally, because CBP has been shown to be an effective human primary cell culture supplement, exchanging CBP for standard cord blood stem cell transplant diluents may improve stem cell survival by creating a more viable microenvironment, which may improve the overall effectiveness of the cell infusions [38–41]. To further advance the translation of hUCB+P therapy for clinical applications, future studies should examine the timing, dose, and route of delivery (e.g., direct injections into the gut) that will optimally sequester inflammation and reduce neurotoxicity [73, 74], altogether retarding the disease progression.

In summary, the present study demonstrated that repeated hUCB+P injections attenuated motor and non-motor deficits in the 6-OHDA rat model of PD. A decrease in neurotoxicity and inflammation in the brain and gut coincided with suppressed expression of specific damaging microbiota located in the gut. That hUCB+P therapy effectively mitigated PD-like dysfunctions in the brain, as well as enhanced non-motor, gastrointestinal functions such as colonic motility through attenuation of inflammatory gut microbiomes support cell-based regenerative medicine [75–81] that targets the brain-gut axis.

## Data Availability

All data reported herein are stored in the Department of Neurosurgery and Brain Repair at the University of South Florida Morsani College of Medicine, and will be available upon request with appropriate end-user agreement.
