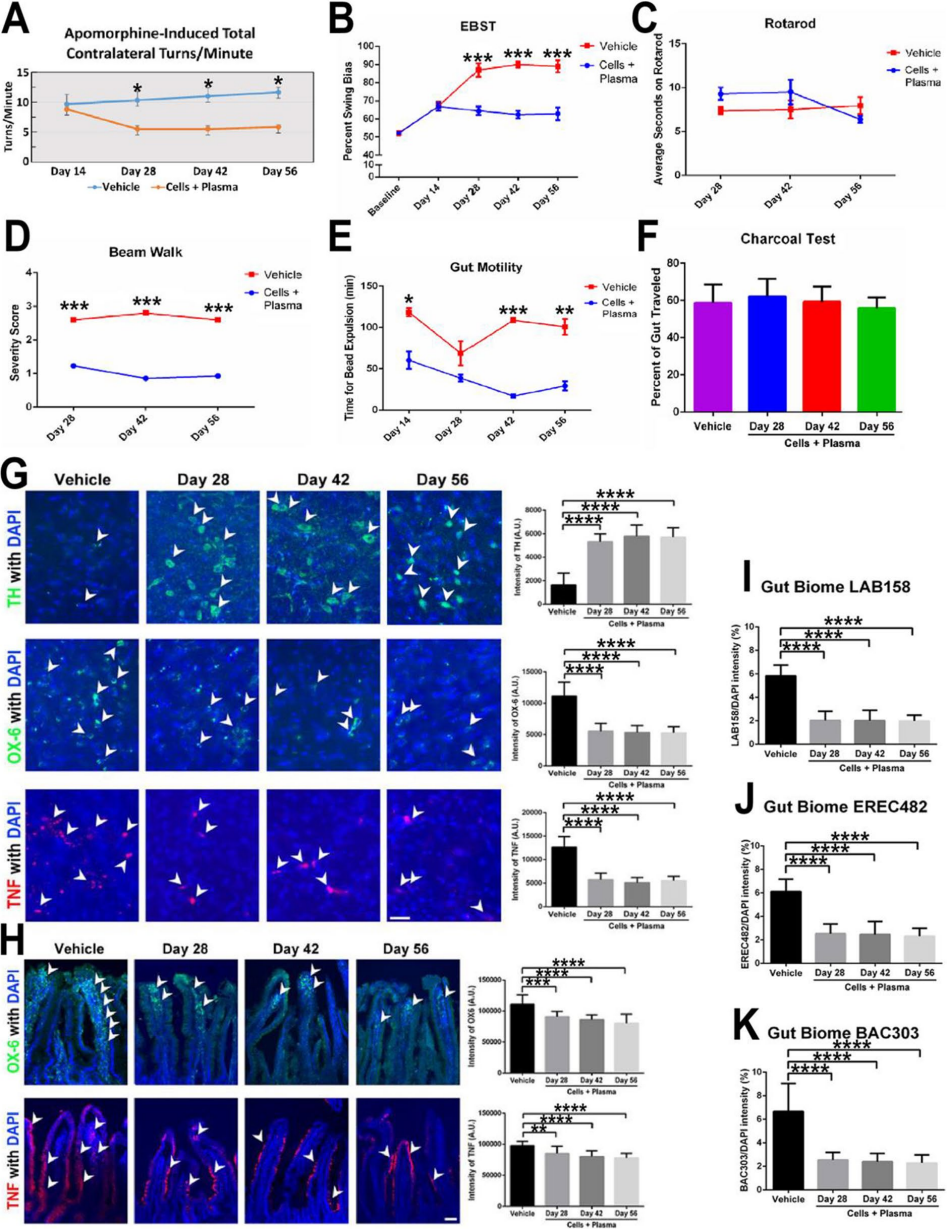# Correction to: A Gutsy Move for Cell-Based Regenerative Medicine in Parkinson’s Disease: Targeting the Gut Microbiome to Sequester Inflammation and Neurotoxicity

**DOI:** 10.1007/s12015-022-10396-y

**Published:** 2022-06-07

**Authors:** Jea-Young Lee, Julian P. Tuazon, Sydney Corey, Brooke Bonsack, Sandra Acosta, Jared Ehrhart, Paul R. Sanberg, Cesario V. Borlongan

**Affiliations:** 1 Center of Excellence for Aging and Brain Repair, Morsani College of Medicine, University of South Florida, 12901 Bruce B. Downs Blvd. MDC 78, Tampa, FL 33612 USA; 2 Department of Neurosurgery and Brain Repair, Morsani College of Medicine, University of South Florida, 12901 Bruce B. Downs Blvd. MDC 78, Tampa, FL 33612 USA; 3 Saneron CCEL Therapeutics, Inc., Tampa, FL 33618 USA; 4 Department of Pathology and Cell Biology, Morsani College of Medicine, University of South Florida, Tampa, FL 33612 USA; 5 Department of Psychiatry, Morsani College of Medicine, University of South Florida, Tampa, FL 33612 USA

**Correction to** **:** **Stem Cell Rev and Rep (2019) 15:690**

**https://doi.org/10.1007/s12015-019-09906-2**

We confirm that an unintentional human error occurred in our paper (Lee JY, Tuazon JP, Corey S, Bonsack B, Acosta S, Ehrhart J, Sanberg PR, Borlongan CV. A Gutsy Move for Cell-Based Regenerative Medicine in Parkinson's Disease: Targeting the Gut Microbiome to Sequester Inflammation and Neurotoxicity. Stem Cell Rev Rep. 2019 Oct;15(5):690-702. doi: 10.1007/s12015-019-09906-2. PMID: 31317505; PMCID: PMC6731204) contained a wrong panel in Figure 5 (panel G, Day 56, upper righthand, and labeled as TH with DAPI). The authors have revisited all the data from this study and we confirm that the reported quantitative data are correct and do not change the results, interpretations, and conclusions of the study. Below is the corrected Figure 5.